# Channeled PM_10_, PM_2.5_ and PM_1_ Emission Factors Associated with the Ceramic Process and Abatement Technologies

**DOI:** 10.3390/ijerph19159652

**Published:** 2022-08-05

**Authors:** Irina Celades, Vicenta Sanfelix, Ana López-Lilao, Salvador Gomar, Alberto Escrig, Eliseo Monfort, Xavier Querol

**Affiliations:** 1 Institute of Ceramic Technology (ITC-AICE), University Jaume I, Campus Universitario Riu Sec, Av. Vicent Sos Baynat s/n, 12006 Castellón, Spain; 2 Institute of Environmental Assessment and Water Research (IDAEA-CSIC), C/Jordi Girona 18, 08034 Barcelona, Spain

**Keywords:** channeled emission, emission factor, particulate matter, abatement technology, ceramic industry, PM_10_, PM_2.5_, and PM_1_

## Abstract

A sampling methodology and a mathematical data treatment were developed that enable to determine not only total suspended particulates (TSP) emitted at channeled sources but also the PM_10_, PM_2.5_, and PM_1_ mass fractions (w_10_, w_2.5_, and w_1_) and emission factors (E.F.), using a seven-stage cascade impactor. Moreover, a chemical analysis was performed to identify the elements present in these emissions. The proposed methodology was applied to different stages of the ceramic process, including ambient temperature (milling, shaping, glazing) and medium–high-temperature (spray-drying, drying, firing, and frit melting) stages. In total, more than 100 measurements were performed (pilot scale and industrial scale), which leads to a measurement time of 1500 h. Related to the mass fractions, in general, the mean values of w_10_ after the fabric filters operated at high performance are high and with little dispersion (75–85%), and it is also observed that they are practically independent of the stage considered, i.e., they are not significantly dependent on the initial PSD of the stream to be treated. In the case of the fine fraction w_2.5_, the behavior is more complex (w_2.5_: 30–60%), probably because the only variable is not the cleaning system, but also the nature of the processed material. Regarding abatement measures, the use of high-efficiency cleaning systems considerably reduces the emission factors obtained for fractions PM_10_, PM_2.5_, and PM_1_. In reference to chemical analysis, the presence of ZrO_2_ and Ni in the spray-drying and pressing stages, the significant concentration of ZrO_2_ in the glazing stage, the presence of Pb, As, and Zn in the firing stage, and the presence of Zn, Pb, Cd, and As compounds in the frits manufacturing should all be highlighted. Nevertheless, it should be pointed out that the use of some compounds, such as cadmium and lead, has been very limited in the last years and, therefore, presumably, the presence of these elements in the emissions should have been also reduced in the same way.

## 1. Introduction

Air pollution has been recognized as the single biggest environmental threat to human health, based on its notable contribution to disease burden [1]. In this sense, European air quality regulations related to particulate matter (hereinafter PM) have been established and significantly modified in the last decades. In the 1990s, only total suspended particles (TSP) were regulated. However, since Directive 1999/30/EC entered into force, limit values for PM_10_ (particulate matter which passes through a size selective inlet with a 50% efficiency cut-off at 10 µm aerodynamic diameter) have been established, specifically, an annual limit value of 40 µg PM_10_ µg/m^3^ with a maximum of 35 days of exceedances of the daily limit value of 50 µg/m^3^. This PM_10_ can be divided into two different categories: coarse fraction, which is mainly deposited in the tracheobronchial region (2.5–10 µm), and fine fraction (<2.5 µm, including ultrafine particles (<0.1 µm)), which can penetrate deep into the lungs and translocate to the other parts of the body [2]. In this regard, in the last years, epidemiologic studies [3,4,5,6] have evidenced the negative effect of fine fraction on health. For this reason, in 2008, Directive 2008/50/EC added PM_2.5_ fraction (particulate matter which passes through a size-selective inlet with a 50% efficiency cut-off at 2.5 µm aerodynamic diameter) and set an annual target value of 25 µg/m^3^. 

In the same line, the recent publication in 2021 of the document WHO Global Air Quality Guidance [1] promotes the necessity to reduce the limit values because of their impact on health. In this regard, this document defines quantitative health-based recommendations for air quality, expressed as either long- or short-term concentrations of different key air pollutants. Specifically, Air Quality Annual Guidance levels of 15 µg/m^3^ and 5 µg/m^3^ are recommended for PM_10_ and PM_2.5_, respectively. These guidelines are not legally binding standards; however, they provide countries with an evidence-informed tool which they can use to inform legislation and policy.

The main contributors of PM are traffic, natural phenomena, combustion in agriculture, domestic fuel burning, and industry [7]. In fact, the emissions into the air are one of the main environmental impacts from industrial activities. With regard to industrial emissions, as with air quality, regulations are becoming increasingly restrictive in terms of permitted concentrations and have broadened the parameters of interest [8]. This behavior is driven following the enforcement of Industrial Emissions Directive (IED, Directive 2010/75/UE) and Integrated Pollution Prevention and Control (IPPC, Directive 1996/61/EC), where Emission Limit Values associated with Best Available Techniques (BAT-AELs) have been established according to BAT Reference Documents (BREFs). As an example, for the ceramics industry (CER-BREF [9]), a generic BAT-AELs for dust is 30–50 mg/Nm^3^. Nevertheless, it should be highlighted that in the BREFs updated after 2012 (cement, wood, ferrous metals, non-ferrous metals, large combustion plants, glass, and waste incineration [10,11,12,13,14,15,16]), the limits established for dust are becoming much more restrictive (1–20 mg/Nm^3^).

In fact, in the discussion and approval of the recent BREFs, PM_10_ and PM_2.5_ are included as parameters to be monitored, but when deriving BAT-AELs, the limit is only established for TSP including PM_10_ and PM_2.5_ [17]. The PM_10_ parameter has appeared for the first time for emissions, additionally to the usual TSP, in tools derived from the IED, knows as Pollutant Release and Transfer Register in Europe (E-PRTR) and Spain (PRTR-Spain [18]).

On the other hand, PM_2.5_ determination can be deemed essential not only because of its potential impact on health, but also because it allows for the detection of anthropogenic particulate pollutants, excluding crustal particulate interference [19]. However, this parameter alone does not seem to be adequate to quantify the impact of some industries with significant primary particulate emissions (such as those of ceramics [19]). For this reason, it is necessary to obtain accurate information on both fractions (PM_10_ and PM_2.5_) in order to identify the contribution of different particulate matter sources and, therefore, to establish specific measures that allow for the improvement of air quality [20].

**Figure 1:**
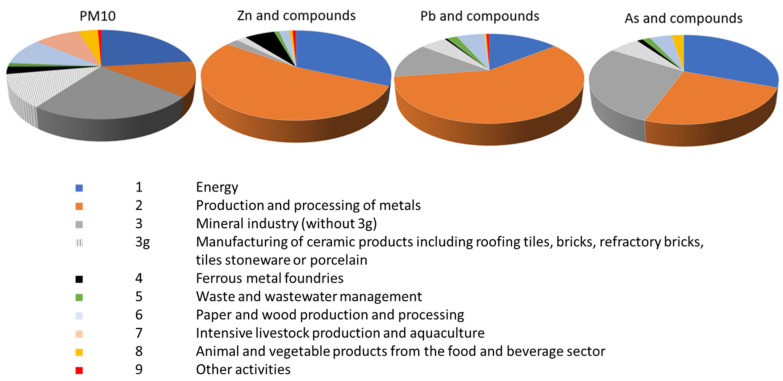
Contribution of ceramic industries (3g) to some air quality indicators in Spain, 2020 [18].

In Spain, according to the values declared in PRTR-Spain, more than 8381.4 tons of industrial primary PM_10_ were emitted into the atmosphere in 2020, of which more than 10% corresponds to ceramic industries [18]. In fact, air quality studies performed in ceramic areas have evidenced the influence of ceramic industry on air quality not only by the presence of high PM concentration, but also for the significant levels of different heavy metals [21,22,23,24]. The contribution of ceramic and related industries to PM_10_ and heavy metals which may be considered as tracers of the ceramic industry [24], information which is available in PRTR, is shown in Figure 1. In Table 1, the main characteristics of ceramic process emissions are shown. 

As it can be drawn for Table 1, the ceramic tile production process may generate both channeled and diffuse emissions:

- Diffuse emissions are those which pass to the atmosphere without being channeled. In the ceramic industry, they are mainly related to bulk material storage, handling, and transport. They can also occur in some operations such as milling, grinding, and trucking.
- Channeled emissions are those which pass to the atmosphere through a pipe. They can be divided into medium–high- and ambient-temperature emissions. The main measures proposed in the CER-BREF [9] to reduce this type of emissions are (1) primary measures: related to reducing the use of raw materials that could contain hazardous components; and (2) secondary measures: different abatement technologies are available as wet scrubber systems, Venturi type, fabric filters, and electrostatic precipitators.

Although the impact of the ceramic industry on air quality is well known [21,22,23,24], PM emission factors related with channeled emissions from ceramic process are not available in the EMEP/EEA air pollutant emission inventory guidebook 2019 [25]. There are some reference documents [26] which are based on US-EPA (Environmental Protection Agency) documents and previous studies [27,28,29,30,31,32,33,34,35,36,37,38,39,40,41,42,43,44,45,46] in which we can check on emission factors for spray-drying, drying, glazing, and firing resulting in a global emission factor between 2.4–11.1 kg TSP/ton depending on the implemented mitigation measures. Nevertheless, these emission factors are only available for TSP. In order to extend the information in public inventories such as E-PRTR and harmonize the key control parameters between air quality and industrial emissions (TSP, PM_10_, and PM_2.5_), the rationale of the present study was, firstly, to develop a sampling methodology based on the previous study performed by Erlich et al. [47,48] and other previous studies performed at industrial scale [49,50,51,52,53,54,55]; secondly, to determine PM_10_, PM_2.5_, and PM_1_ emission factors associated with different stages that take place during the ceramic tiles manufacturing process.

**Figure 2:**
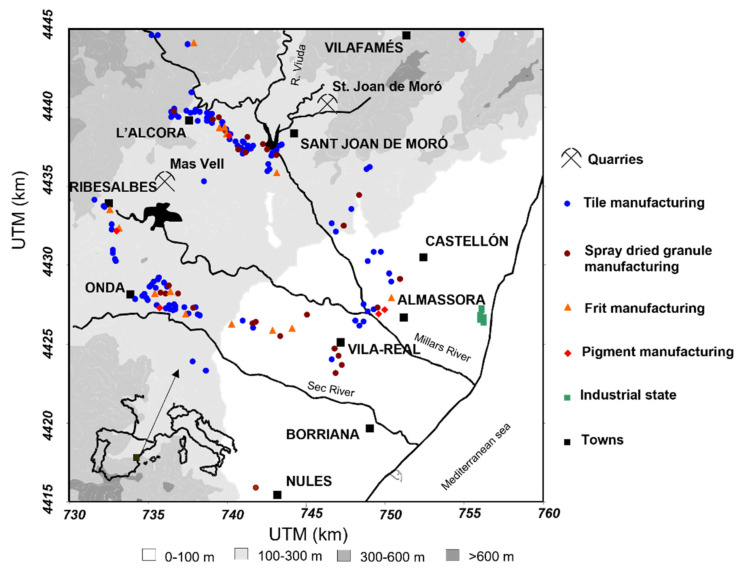
Map of the Castelló ceramic cluster [59].

To this aim, this study was performed in a wide variety of facilities located in the ceramic production area of Castelló (Figure 2). This area extends from the coastal flat (mainly occupied by residential areas and orange tree plantations) to the mountain chain of La Cruz. This area is the largest ceramic-tile-producing zone in the EU, accounting for a turnover in 2020 of approximately EUR 3842M [56] and EUR 1200M [57] for ceramic tiles and frit and pigments, respectively. As a consequence of the high concentration of ceramic and related industries in a small area, an Air Quality Plan [58] was elaborated in 2008 to implement high-efficiency PM emissions abatement technologies in ceramic facilities and to replace impurity-bearing raw materials.

## 2. Methodology

The method to perform the PM monitoring campaigns is based on the inertial separation of the PM target fractions and its subsequent gravimetric determination. The applied methodology was performed in accordance with the reference standards [60,61] and the specific previous studies focused on ceramic emissions [62,63], and its application allowed us to obtain the chemical characterization of the ceramic PM channeled emissions. 

### 2.1. Physical Characterization of the PM Emitted

The physical characterization allowed for the determination of particle size distribution (PSD), mass fractions (w_x_: w_10_, w_2.5_, and w_1_) and specific emission factors (EF_10_, EF_2.5_, and EF_1_) for each process stage. Measurement campaigns were carried out at several ceramic companies which manufacture wall and floor tiles and frits (Table 2). All measurements were carried out under real operating conditions. The sampling period was chosen in such a way that sufficient mass is collected to permit weighing with the required accuracy without overloading the stages. Since the total PM concentrations were usually low at the tested industrial plants, very long sampling times must be provided for the reasons mentioned.

It should be highlighted that, in some stages, measures were performed before and after the cleaning system (cyclone, wet scrubber, fabric filter, and electrostatic precipitator).

With this aim, experimental measures were taken at industrial scale using a cascade impactor (Anderson Impactor type Mark III). This impactor is designed to meet the specifications reported by VDI 2066 [64,65] and to fractionate suspended particles into different sizes categories according to their inertia. 

The cut-size associated with each impactor stage depends on flow and temperature of the airstream. To calculate the PSD and mass fractions of interest (w_10_, w_2.5_, and w_1_) easily and accurately, the results need to be adjusted to a distribution. This distribution could be the log-normal one, which is the most usual to treat PSD data because, from the mathematical point of view, it ensures that all obtained values are positive and, therefore, they have a physical meaning. From the literature review, different types of distributions has been identified, which yield very good results in this field, such as the Rosin–Rammler–Sperling–Bennet distribution (RRSB) [47,66]. For this reason, the results obtained with log-normal distribution in the present study were compared with those obtained by RRSB distribution.

**Figure 3:**
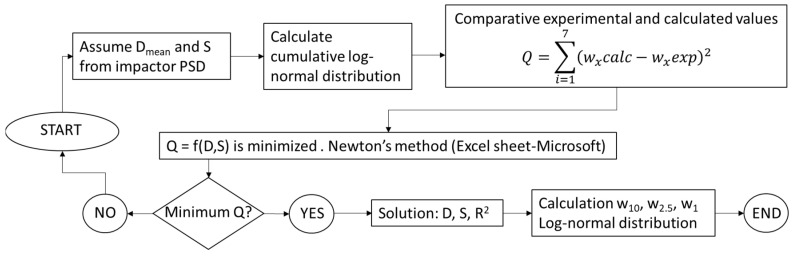
Flow chart of the mathematical treatment used to calculate PSD and w_x_. D: geometric diameter (µm); S: geometric deviation; R: correlation index.

The procedure applied to calculate the cumulative log-normal and RRSB distribution is described in Figure 3.

The detailed assessment of the proposed methodology was carried out in previous studies [62,63]. The evaluation criteria used was the compatibility index (CI) (EN ISO/IEC 17043 [67]), which allows us to know if two results associated with their respective uncertainties are comparable. The results compared were the total concentration measurement determined by using a cascade impactor and one obtained with the standard method (EN 13284-1 [68]). The CI was satisfactory in most of the cases studied, so it was considered that there was a good correlation between the compared concentrations. Therefore, both distributions can be considered appropriate for the objectives of the present study.

Despite the favorable results obtained in this study, and following the recommendations of the standards (EN ISO 23210 [69]), the use of a cascade impactor for the quantification of the total PM concentration is not recommended in those cases where the objective of the measurement is to ensure compliance with the established regulations. 

### 2.2. Chemical Characterization

The objective of the chemical characterization was to obtain the chemical profile of the mass fractions w_10_ and w_2.5_ from the emissions associated with the ceramic process.

The selection of the analysis technique for the determination of the mass concentration of specific elements in the particulate matter emissions of the studied industrial processes was dependent on the amount of sample required to perform the analysis. In fact, it was the main drawback of samplings carried out at industrial scale.

**Figure 4:**
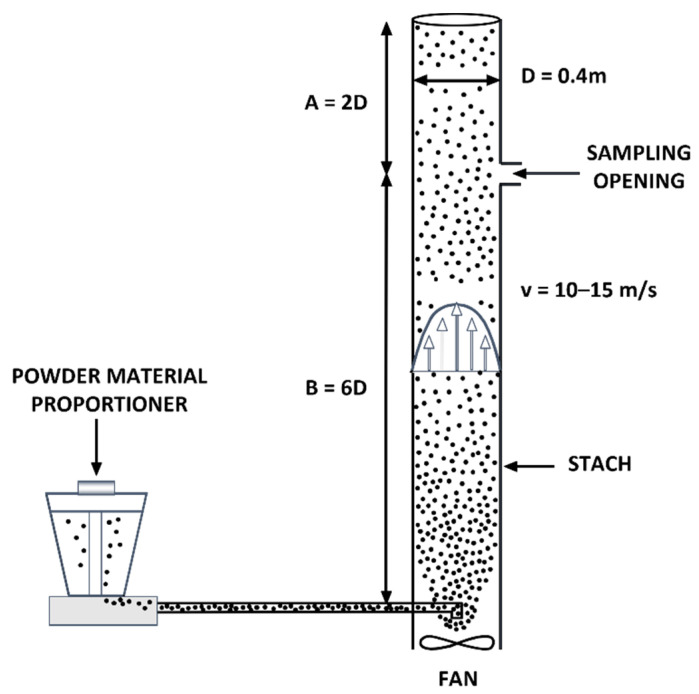
PM emissions generator diagram.

This situation is more critical because of the extensive implementation of Best Available Techniques in the ceramic plants, which significantly reduces the emissions and requires very long sampling times, which makes it difficult to comply with the technical criteria established in the sampling standards. In these cases, the measurements were carried on a pilot scale (emission simulator; Figure 4), whose use was evaluated in previous studies [62,63].

The PM emissions generator allows for the regulation of the flow rate and the amount of solid material fed into an airstream with an air velocity similar to industrial installations (10–15 m/s) which transfer the dust to the sampling area, obtaining a range of PM concentrations. The feasibility of this system was deemed essential to obtain enough amount of sample for chemical analysis, upholding the technical requirements of the sampling standards. In this system, the powdered material used was provided by ceramic industries from the waste captured by the fabric filters installed to abate PM emissions generated in each stage of the process (Table 2).

The sampling by means of the PM emissions generator is based on the assumption that the content of PST and fractions w_10_, w_2.5_, and w_1_ of the material collected by the different cleaning systems (fabric filter and electrostatic precipitator) is similar to the composition of the w_10_, w_2.5_, and w_1_ of the emissions generated after the abatement system. This assumption is based on the fact that the temperature of the gases as they pass through the cleaning systems is of the same order as the emission temperature, and therefore, a priori, it is not expected that condensation processes, which could modify the chemical composition of the issued PM, take place.

The device used for collecting the sample was a Tecora cyclone, designed to meet the specifications reported by USEPA in the Method 201A [70] and to measure PM_10_ and PM_2.5_ in stack emission [62,63]. This device allowed us to obtain the required amount of sample to subsequently perform the chemical analysis of the PM_10_ and PM_2.5_ captured. The cyclone is required since the cascade impactor has different stages and, therefore, it would be very difficult to obtain enough mass of sample for the subsequent analysis using this device.

The sampling of particulate matter was carried out by means of quartz glass filters (QF20 Schleicher and Schuell). Once the PM concentrations were obtained by weighting the filters using standard procedures, one-half of each of them was digested and analyzed following the method by Querol et al. [19,71]. This method is based on an acid attack using low-pressure Teflon bombs. The solution obtained was then centrifuged and analyzed by (a) inductively coupled plasma–atomic emission spectrometry (ICP-AES) for major elements, and (b) inductively coupled plasma–mass spectrometry (ICP-MS). A quarter of each filter was used to analyze boron by the Azomethine-H method. Finally, the last quarter was sometimes used for the morphological characterization. 

### 2.3. Summary of Sampling Campaigns

In total, more than 100 measurements were performed (pilot scale and industrial scale), which led to a measurement time of 1500 h (Table 3).

## 3. Results

### 3.1. Physical Characterization of the PM Emissions

#### 3.1.1. Determination of PSD and w_x_

**Figure 5:**
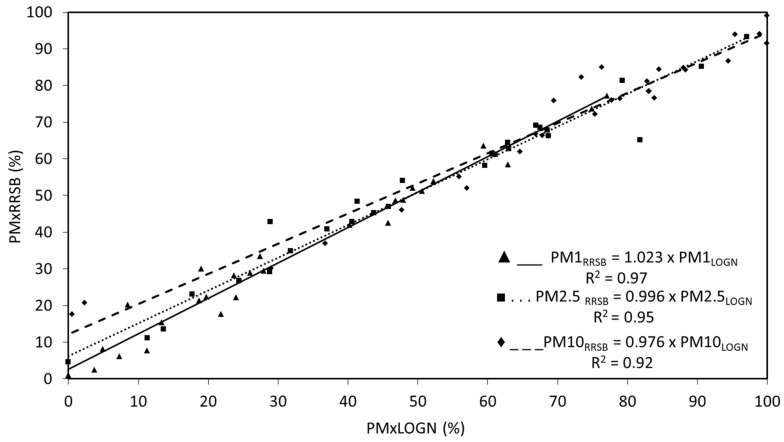
Granulometric fractions w_10_, w_2.5_, and w_1_ calculated by the log-normal method and RRSB method.

The determination of w_x_ was obtained from the PSD, applying the mathematical treatment described in Figure 3. In this regard, two mathematical methods were evaluated: log-normal and RRSB (Section 2.1). It can be observed that both methods are comparable in all cases (Figure 5), so the log-normal model was applied, since it is the commonly used one in the surveyed literature.

The average fractions w_10_, w_2.5_, and w_1_ obtained from the PSD are shown in Table 4 and Table 5. More detailed information about the average PSD is shown in the Supplementary Material (Figures S1–S3 and Tables S1 and S2). The average process stage PSD was calculated from the sum of the mass of the particles (within the same size range) of each of the individual samplings.

In order to compare the results, graphs representing the cumulative probability (cumulative mass expressed in %) versus particle diameter were produced (Figures S1–S3). This type of graph is easy to interpret and yields a straight line whenever the characterized particles come from a single source or when they have similar sizes, even if they come from several sources [72,73,74].

The average w_x_ (expressed in %), as a function of the particle size, was grouped attending to the following criteria, to be easily understood:

- Emissions generated in ambient-temperature processes (<50 °C): milling, pressing, and glaze preparation and glazing (Table 4).
- Emissions generated in medium–high-temperature processes (60 °C to >150 °C): drying, spray-drying, firing, and frits melting (Table 5).

It is remarkable the long sampling times (>40 h) required to determine the w_x_ and PSD in those stack emissions with low particle concentrations (dryers or emissions after treatment).

#### 3.1.2. Determination of EF

This section sets out the specific emission factors obtained for the PM_10_, PM_2.5_, and PM_1_ for the different stages of the ceramic process, expressed as mgPMx/m^2^ or mgPMx/kg depending on the characteristics of the processed product. For this purpose, a specific weight of 21 kg of spray-dried granulate/m^2^ was considered and the specific flow rates were obtained from previous studies in the ceramic industry [58,59].

The sources studied were divided into two groups, based on process temperature, as was performed in Section 3.1.2, since the literature reports and results obtained from the present study evidenced that this characteristic notably influences the size and composition of the PM emitted from these sources. Emission factors are shown in Table 6 and Table 7.

### 3.2. Chemical Composition of PM Emissions 

In this section, the average chemical profiles are shown (Table 8), including major and trace elements of PM emission from ceramic process stages. This average profile was obtained from at least three individual valid samplings for each process stage. The major elements (expressed as oxides) are those whose percentage in composition is higher than 1%, and the trace elements are those where the concentration is higher than 100 mg/kg.

In stages such as spray-drying, pressing, and drying, where the processed product is quite similar (in terms of chemical composition) and has not suffered any significant physical–chemical transformations (low–medium process temperature), it is considered that the chemical profile is common for the different stages. More detailed information about the identified major and trace elements can be obtained from the Supplementary Material (Figures S4–S9).

**Figure 6:**
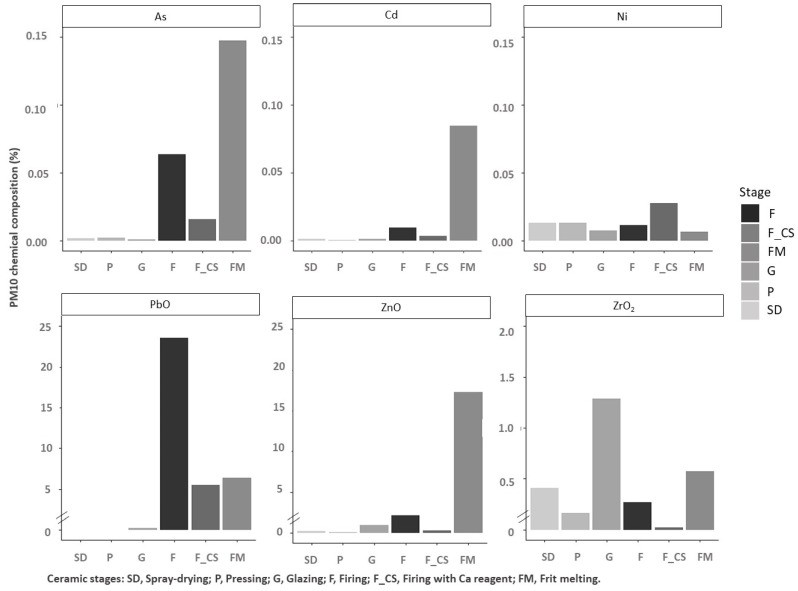
PM_10_ composition in the different stages of the ceramic process.

**Figure 7:**
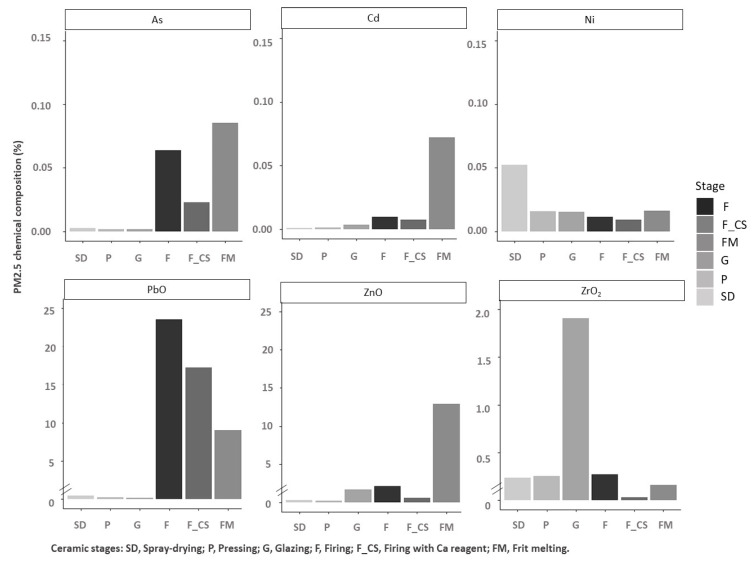
PM_2.5_ composition in the different stages of ceramic process.

From these results (Table 8), and taking into account previous air quality studies performed in the ceramic area of Castellón, the main tracers and other legislative elements (Ni and Cd) were selected to study the segregation of these elements in fractions PM_10_ (Figure 6) and PM_2.5_ (Figure 7), associated with the different stages of ceramic process considered.

## 4. Discussion

The Discussion section follows the same structure as the Results section.

### 4.1. Physical Characterization of the Emitted PM

#### 4.1.1. Determination of PSD and w_x_

Regarding the comparison between the log-normal and RRSB distributions, it can be seen that both methods are comparable since in all cases (w_10_, w_2.5_, and w_1_) a trend line with a slope coefficient close to 1 and a regression coefficient higher than 0.90 was obtained. In addition, none of the methods have a clear tendency to either overestimate or underestimate the results. It can therefore be concluded, considering this evaluation, that both methods can be indistinctly used, so the log-normal model was applied, since it is the most commonly used one in the surveyed literature.

In reference to the PSD obtained in the present study, the fit parameters to a log-normal distribution, obtained by applying the calculation procedure described in Figure 3 for the calculation of w_x_, showed good agreement (R^2^ > 0.90).

**Figure 8:**
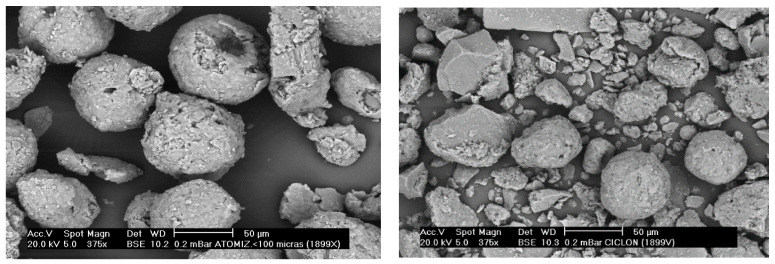
SEM photograph of the PM emitted by the cyclones after spray-drying.

**Figure 9:**
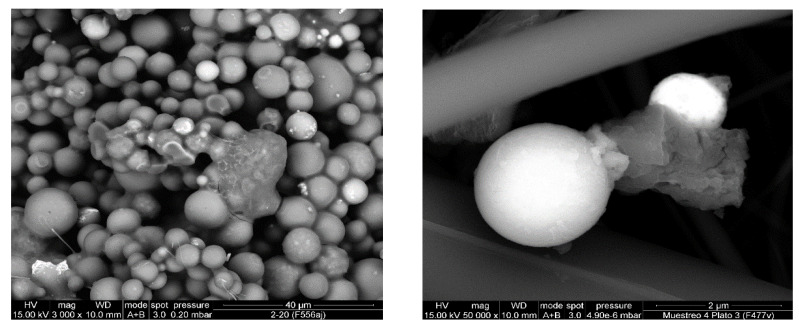
SEM photograph of the PM emitted in the frit melting stage.

From the results obtained without cleaning system, wide PSD can be observed. In the case of ambient- and medium-temperature process stages, it can be due to the presence of coarse particles, in the form of granulates and agglomerates, and fine particles. The fine particles are associated with individual particles of the processed material and particles generated by the breakage of agglomerates/aggregates (Figure 8). In high-temperature processes, wide PSD is also observed as a result of different origins for PM, coarser material generated by carryover of batch particles and finer PM from volatilization–condensation processes (Figure 9).

Regarding the influence of the cleaning systems on the particle size of the emitted PM, it should be noted that, in most of the processes studied (except the firing stage with solid reagent injected in the exhaust stream to remove gaseous pollutants), the finest fractions are enriched after the cleaning system. Moreover, the PSDs were less wide, presumably due to the fact that the coarser fraction was highly efficiently reduced. Finally, it was considered interesting to make some specific comments on some of the stages and cleaning systems studied (Table 9).

In general, the mean values of w_10_ after the fabric filters operated at high performance are high and with little dispersion (75–85%), and it is also observed that they are practically independent of the stage considered, i.e., they are not significantly dependent on the initial PSD of the stream to be treated.

In the fine fraction w_2.5_, the behavior is more complex (w_2.5_: 30–60%), probably because the main variable is not the cleaning system, but also the nature of the processed material.

#### 4.1.2. Determination of EF

In general, the use of high-efficiency cleaning systems considerably reduces the emission factors obtained for fractions w_10_, w_2.5_, and w_1_. Nevertheless, the type of cleaning system and other operational parameters (such as the material processed and/or process temperature) can have an influence on the obtained results:

- In medium–high-temperature stages, concretely in the spray-drying and frits melting, the emission factors obtained differ by a factor of 10 in the case of frit melting and by a factor of 100 in the case of spray-drying, depending on the Best Available Technique (BAT) implemented. The lowest values correspond to the technological scenario corresponding to fabric filters.
- In those stages where the processed materials are similar, but the process temperature is significantly different, such as the milling and spray-drying stages, the average emission factors obtained were very similar when a fabric filter was used as an abatement technology (EF_PM10_: 2–3 mg/kg, EF_PM2.5_: 2 mg/kg, and EF_PM1_: 1 mg/kg).
- On the contrary, when the material processed is different and the process temperature is similar, such as the pressing and glazing stages, the emission factors obtained differ considerably (EF_PM10_: 8–48 mg/m^2^, EF_PM2.5_: 3–31 mg/m^2^, and EF_PM1_: 1–29 mg/m^2^).

The obtention of specific emission factors for different particle size and stage processes, including the influence of the abatement system, is considered of great practical interest for the ceramic industry, technological providers, public authorities, and research groups for performing emission inventories (e.g., E-PRTR), deriving new BAT-AELs (Emission Limit Values associated with Best Available Techniques), air pollution diagnosis studies, environmental impact studies from a lifecycle analysis perspective, and air quality assessment studies, among others.

Despite the potential use of the ceramic specific emission factors mentioned above, it is remarkable that these are not currently available in those reference emission factors guides such as the AP-42/EPA [26] and EMEP-EEA [25] and in the BAT Reference Document applicable to the ceramic and related industries (CER BREF [9], GLS BREF [12], and WGC BREF [17]).

Table 10 shows a compilation of emission factors obtained in other similar studies [47] and in the present study. In general, they are coherent PM emissions from combustion processes (e.g., firing and fusing) finer than those generated from mechanical treatments (e.g., press and milling). In one case, significant deviations were detected between comparable processes, such as isostatic pressing of minerals; this could be due to the different sizes of the material processed.

### 4.2. Chemical Characterization of the PM Emissions

Finally, regarding the chemical analysis, the following conclusions may be drawn:

- In the spray-drying and pressing stages there was an important presence of ZrO_2_ and Ni, which can be attributed to the presence of these components in the raw materials and also in the ceramic sludges. It should be highlighted that the ceramic sludges, which are reused in the spray-drying stage, are generated during different cleaning operations (glaze preparation and glazing stage) and, therefore, they have a similar composition to the raw materials used in these last-mentioned processes.
- In the case of glazing, the concentration of ZrO_2_, which is mainly used to achieve opacity, is significant. Nevertheless, this composition can be very variable because it depends on the final aesthetic requirements of the ceramic tiles produced, and this compound is not present in all glaze compositions.
- Regarding the firing stage (emissions before the abatement system), the presence of PbO, As, and Zn compounds was associated with the raw materials used in body and glaze composition.
- Finally, in the case of the melting stage for frits manufacturing, the presence of Zn, PbO, Cd, and As compounds was linked to raw material compositions. Nevertheless, it should be highlighted that the use of cadmium and lead in the frits composition has been very limited in the last years, and, therefore, presumably the presence of these elements in the emissions should have been reduced in the same way.

Another aspect studied was the possible segregation of the components and elements of interest (As, Cd, Ni, PbO, ZnO, and ZrO_2_) in the PM_10_ and PM_2.5_ fractions for each of the process stages. It was observed that the enrichment in one or another fraction is associated with the emission mechanism of the component and/or element evaluated and with the granulometry of the source material.

- For example, in the spray-drying stage, there is an enrichment in Ni in the PM_2.5_ fraction. The presence of this element is associated with the use of ceramic pigments whose granulometry is usually very fine.
- Regarding the emission mechanism, the fluxing nature of lead compounds means that this is present mostly by volatilization from the melt; hence, an enrichment of this component is observed in the finest fraction, specifically in the frit melting and firing stages after the cleaning system.
- In the melting stage of ceramic frits, both As, a trace element associated with the natural raw materials that introduce boron into the composition of ceramic frits, and ZrO_2_, a raw material for frits and atomized granules, are enriched in the PM_10_ fraction, probably because the emission mechanism in both cases is mechanical in nature.

## 5. Conclusions

The conclusions of this study are presented in accordance with the structure followed in the previous sections.

### 5.1. Physical Characterization

#### 5.1.1. Assessment of Methodology Used to Determine w_x_ and PSD

- The cascade impactor is suitable for determining and studying both the PSD and the w_10_, w_2.5_, and w_1_ fractions. The use of the impactor is not recommended due to the uncertainty that may be associated with the filters weighted.
- The two mathematical processing methods of the PSD data (log-normal distribution and Rosin–Rammler–Sperling–Bennet) exhibit similar results for the determination of the w_10_, w_2.5_, and w_1_ fractions. The log-normal adjustments with R^2^ greater than 0.90 were obtained.

#### 5.1.2. Determination of PSD and w_x_

- The PSDs obtained are relatively wide, due to the different mechanisms of origin of the particulate matter, mechanical, and/or volatilization–condensation.
- The average values obtained for w_10_ and w_2.5_ after fabric filters, operated at high performance, are in the range of 75–85% and 30–60%, respectively. In the case of the fine fractions, the wide range is due to the influence not only of the cleaning system but also of the nature of the processed material.

#### 5.1.3. Determination of the EF

- The highest efficiency, for the ceramic stages studied, is reached when fabric filters are applied.
- In those cases where the materials processed are similar, such as the milling and spray-drying stages, the process temperature does not significantly affect the EFs obtained.

### 5.2. Chemical Characterization of the PM Emissions

- The use of the pilot-scale emission simulator made it possible to significantly reduce the sampling times and consequently to obtain a large amount of samples of the different granulometric fractions, which allowed a complete chemical characterization.
- The chemical analysis showed that ZrO_2_, ZnO, BaO, PbO, B_2_O_3_, Hf, Cr, Cu, Cd, and Sn were the main components in the emissions related to ambient-temperature processes (spray-drying, pressing, and glazing) while SiO_2_, Al_2_O_3_, CaO, MgO, Na_2_O, K_2_O, BaO, ZnO, PbO, S, Tl, As, Cr, Rb, Cs, Cu, and Sr were the main components of the medium–high-temperature processes emissions (firing and frits melting).
- The emission mechanism (mechanical and/or volatilization–condensation) and the particle size of the source material are the parameters that most influence the potential segregation of the components and elements evaluated (As, Cd, Ni, PbO, ZnO, and ZrO_2_) in the PM_10_ and PM_2.5_ fractions.

## 6. Future Research Lines

From the results obtained in the present study, a series of future research lines to complement some of the results achieved are proposed:

- To develop an emission simulator in order to modify the temperature of the stream and the introduction of gases, and thus study the effect of the temperature and the composition of the gas stream on the characteristics of the particulate matter.
- To use more complete particle size distribution determination systems, even complementing several systems simultaneously, which allow us to obtain information in a wider range of particle sizes. In this sense, it would be especially interesting to address the study of the submicron and ultrafine fractions in emissions from high-temperature processes, both in concentrations and their variation and possible correlation with emission mechanisms and scrubbing systems. These fractions are of increasing environmental interest, and therefore receive greater legislative attention due to their possible effects on health.
- It would be extremely interesting to initiate a line in collaboration with analytical chemists to clarify some aspects that could not be determined in this work, such as the detailed study of the behavior of some elements and their compounds, in particular of the most volatile elements, such as boron, lead, arsenic, thallium, etc., in processes and emissions at higher temperatures, in order to deepen the knowledge of the volatilization–condensation mechanisms and minimize possible emissions. To this end, it is necessary to develop methodologies that integrate equipment allowing for the characterization of very small samples.
- To carry out a specific study of high-temperature process steps in order to determine the influence of raw material composition, process variables, scrubbing systems, etc., on the characteristics of particulate matter emissions.
- To complement the results of this study with air quality studies in the area, for which it is necessary to determine the behavior of particulate matter and gases emitted by different sources in the atmospheric environment, studying the mechanisms of interaction between different primary and secondary pollutants.

## Figures and Tables

**Table 1:** Ceramic process emissions.

Process	Stage	Emission	Flow	Type	Pollutant
Tiles	Storage and handling of raw materials	Variable	Continuous	Diffuse	PM
	Milling (dry)	Variable	Continuous/Discontinuous	Ambient channeled	PM
	Milling (wet)				
	Spray-dried	Constant	Continuous	^1^ Hot channeled	PM and gases
	Pressing	Variable	Continuous	Ambient channeled	PM
	Dry	Constant	Continuous	^1^ Hot channeled	PM and gases
	Glaze preparation	Variable	Discontinuous	Ambient channeled	PM
	Glazing	Variable	Continuous	Ambient channeled	PM
	Firing	Constant	Continuous	^1^ Hot channeled	PM and gases
Frits	Milling	Variable	Continuous/Discontinuous	Ambient channeled	PM
	Frits melting	Constant	Continuous/Discontinuous	^1^ Hot channeled	PM and gases

^1^ Hot channeled refers to those medium–high-temperature processes.

**Table 2:** Industrial scenarios.

Industrial Process	Stage Process
Ceramic tile	Milling
	Spray-drying
	Pressing
	Glaze preparation and glazing
	Drying
	Firing
Ceramic frit	Frit melting

**Table 3:** Sampling campaigns description.

	Physical Characterization	Chemical Characterization
Scenario	Industrial	Pilot scale (PM emission generator)
Device	Cascade impactor	PM_10_/PM_2.5_ cyclone
Sampling campaigns	Number of samplings: 47 Sampling hours: 1150	Number of samplings: 95 Sampling hours: 470

**Table 4:** w_10_, w_2.5_, and w_1_ obtained during milling, pressing, and glazing (ambient emissions).

Process Stage	Cleaning System	Number of Samplings (*n*)	T_gases_ (°C)	Average Values			
				C_TI_ (mg/Nm^3^)	w_10_ (%)	w_2.5_ (%)	w_1_ (%)
Milling	Fabric filter	4	15–30	<5	74.8	53.4	38.0
Pressing	None	1	15–30	109 ± 33	21.0	2.1	0.23
	Fabric filter	1		<5	75.3	28.9	5.3
Glaze preparation and glazing	None	2	18–40	132 ± 71	51.8	20.1	7.5
	Fabric filter	3		<5	74.5	41.7	22.9

**Table 5:** w_10_, w_2.5_, and w_1_ obtained during drying, spray-drying, firing, and frits melting (medium- and high-temperature emissions).

Process Stage	Cleaning System	Number of Samplings (*n*)	T_gases_ (°C)	Average Values			
				C_TI_ (mg/Nm^3^)	w_10_ (%)	w_2.5_ (%)	w_1_ (%)
Spray-drying	Cyclone	1	75–120	>1000	73.4	41.3	21.8
	Fabric filter	2		<5	75.4	50.0	33.2
	Cyclone + wet scrubber	2	60–65	75 ± 34	97.7	80.6	39.2
Drying	None	1	110–120	<5	84.5	66.9	52.2
Firing	None	2	160–210	11 ± 5	99.4	93.9	75.9
	Fabric filter + reagent	2	140–160	<5	81.6	59.2	41.7
Frits melting	None	3	110–260	415 ± 318	74.9	59.1	43.9
	Fabric filter	1	110–210	<5	83.1	61.1	43.5
	Electrostatic precipitator	1		<5	88.0	67.5	49.2

**Table 6:** Emission factors for milling, pressing, and glaze preparation and glazing (ambient-temperature processes).

	Process Stage	Cleaning System	^1^ Q (Nm^3^/kg)	Units	Average Values		
					EF_PM10_	EF_PM2.5_	EF_PM1_
Samplings at ambient temperature sources (T < 40 °C)	Milling	Fabric filter	4	mg/kg	2	2	1
	Pressing	None	4	mg/m^2^	1923	192	21
		Fabric filter			8	3	1
	Glaze preparation and glazing	None	4	mg/m^2^	7183	2212	724
		Fabric filter			48	31	29

^1^ Specific flow rate obtained from Monfort et al., 2013 [59] and Conselleria de Medi Ambient, Aigua, Urbanisme i Habitatge, 2008 [58].

**Table 7:** Emission factors for spray-drying, drying, firing, and frits melting (medium- and high-temperature processes).

	Process Stage	Cleaning System	^1^ Q (Nm^3^/kg)	Units	Average Values		
					EF_PM10_	EF_PM2.5_	EF_PM1_
Samplings at medium–high-temperature ceramic sources	Spray-drying	None	4	mg/kg	4147	2734	1137
		Fabric filter			3	2	1
		Wet scrubber			297	244	99
	Drying	None	3	mg/m^2^	155	123	96
	Firing	None	4	mg/m^2^	901	857	690
		Fabric filter + solid reagent			11	8	5
	Frit melting	None	4.4	mg/kg frit	1376	1045	643
		Fabric filter			1	1	1
		Electrostatic precipitator			19	15	11

^1^ Specific flow rate obtained from Monfort et al., 2013 [59] and Conselleria de Medi Ambient, Aigua, Urbanisme i Habitatge, 2008 [58].

**Table 8:** PM emission composition (major and trace elements) associated with different ceramic process stages.

Process Stage	Major Elements	Trace Elements
Spray-drying Drying Pressing	ZrO_2_, ZnO, BaO, PbO	Hf, Cr
Glazing	B_2_O_3_, BaO, PbO, ZnO, ZrO_2_	Cu, Cr, Cd, Sn, Hf
Firing	Na_2_O, K_2_O, ZnO, PbO	S, Tl, As, Cr, Rb, Cs, Cu
Frits melting	SiO_2_, Al_2_O_3_, CaO, MgO, K_2_O, Na_2_O, BaO, ZnO	S, Sr, Cs

**Table 9:** Comments about w_x_ associated with different ceramic process stages and cleaning systems.

Process Stage	Cleaning System	Granulometry
Spray-drying	Cyclone + wet scrubbing system	The increase of w_x_ is due to the breakage of the agglomerates by wetting.
Firing	Fabric filter + solid reagent	The reduction of w_x_ is a direct consequence of injecting solid reagent to remove gaseous pollutants.
Frit melting	Fabric filter	The increase of the w_x_ emitted post-cleaning can be explained by the thermal origin (volatilization–condensation) of the particles when the temperature of the exhaust gases is reduced (<200 °C) before entering into the cleaning system.

**Table 10:** Compilation of emission factors.

Source	Industrial Process	Subsector, Basic Input Material, Fuel	Cleaning System	TSP (mg/Nm^3^)	Mean Values (%)		
					EF_PM10_	EF_PM2.5_	EF_PM1_
Findings of this research	Ceramic tile manufacturing	Ceramic tile manufacturing, milling, batch	Fabric filter	<5	74.8	53.4	38.8
Previous studies [47]	Treatment natural stone, sand	Crusher plant, limestone, dolomite	Fabric filter	1.2	69.2	14.2	5.0
Findings of this research	Treatment natural stone, sand	Preparation of ceramic raw materials, loam, clay, porosity material	Fabric filter	0.8	80.4	34.4	16.5
Findings of this research	Ceramic tile manufacturing	Ceramic tile manufacturing, isostatic compression press, spray-dried powder	Fabric filter	<5	75.3	28.9	5.3
Previous studies [47]	Manufacture of porcelain/press	Isostatic compression press, porcelain substance	Fabric filter	0.1	94.9	57.4	38.3
Findings of this research	Ceramic tile manufacturing	Ceramic tile manufacturing, firing, continuous, natural gas	None	11	99.4	93.9	75.9
Previous studies [47]	Tunnel oven ceramic industry	Oven without additive, loam, clay, gas	None	5.3	93.9	85.0	79.7
		Oven with additive, loam, clay, gas, lime	None	3.4	95.4	88.6	84.9
Findings of this research	Glass industry	Ceramic frit manufacturing	Fabric filter	<5	83.1	61.1	43.5
Ehrlich et al. 2007 [47]	Glass industry	Manufacture of goblets and beakers, bath, cullet, batch, natural gas	Fabric filter	0.8	93.4	53.3	37.7

## Supplementary Materials

The following supporting information can be downloaded at https://www.mdpi.com/article/10.3390/ijerph19159652/s1: Figure S1. PSD of emissions generated in ambient temperature processes; Figure S2. PSD of emissions generated in medium-temperature processes; Figure S3. PSD of emissions generated in high-temperature processes; Figure S4. PM_10_ and PM_2.5_ composition of emissions generated during spray-drying emissions; Figure S5. PM_10_ and PM_2.5_ composition of emissions generated during pressing; Figure S6. PM_10_ and PM_2.5_ composition of emissions generated during drying; Figure S7. PM_10_ and PM_2.5_ composition of emissions generated during firing (without cleaning system); Figure S8. PM_10_ and PM_2.5_ composition of emissions generated during firing (after cleaning system); Figure S9. PM_10_ and PM_2.5_ composition of emissions generated during frit melting. Table S1: Individual samplings of ambient temperature processes; Table S2. Individual samplings of medium- and high-temperature processes.
