# Supplementary material for: Channeled PM10, PM2.5 and PM1 Emission Factors Associated with the Ceramic Process and Abatement Technologies
Source: Int J Environ Res Public Health. 2022 Aug 5;19(15):9652. doi: 10.3390/ijerph19159652 (PMC9367777; doi:10.3390/ijerph19159652)
Supplement: Supplementary file 1 [file ijerph-19-09652-s001.zip › ijerph-1712157-supplementary.pdf]

Supporting Information:

Figure S1. PSD of emissions generated in ambient temperature processes

Figure S2. PSD of emissions generated in medium temperature processes

Figure S3. PSD of emissions generated in high temperature processes

Table S1: Individual samplings of ambient temperature processes

Table S2. Individual samplings of medium and high temperature processes

Figure S4. PM10 and PM2.5 composition of emissions generated during spray-drying emissions

Figure S5. PM10 and PM2.5 composition of emissions generated during pressing

Figure S6. PM10 and PM2.5 composition of emissions generated during drying

Figure S7. PM10 and PM2.5 composition of emissions generated during firing (raw emissions)

Figure S8. PM10 and PM2.5 composition of emissions generated during firing (after cleaning system)

Figure S9. PM10 and PM2.5 composition of emissions generated during frit melting

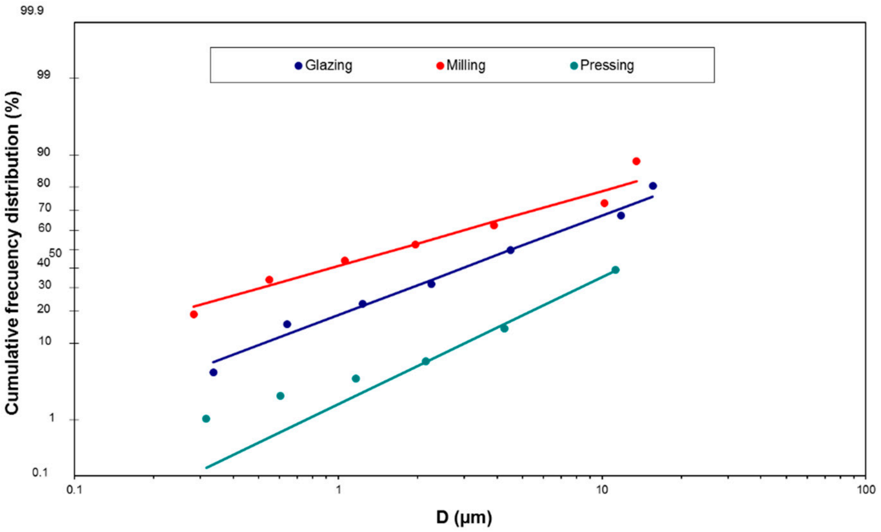

Figure S1. PSD of emissions generated in ambient temperature processes

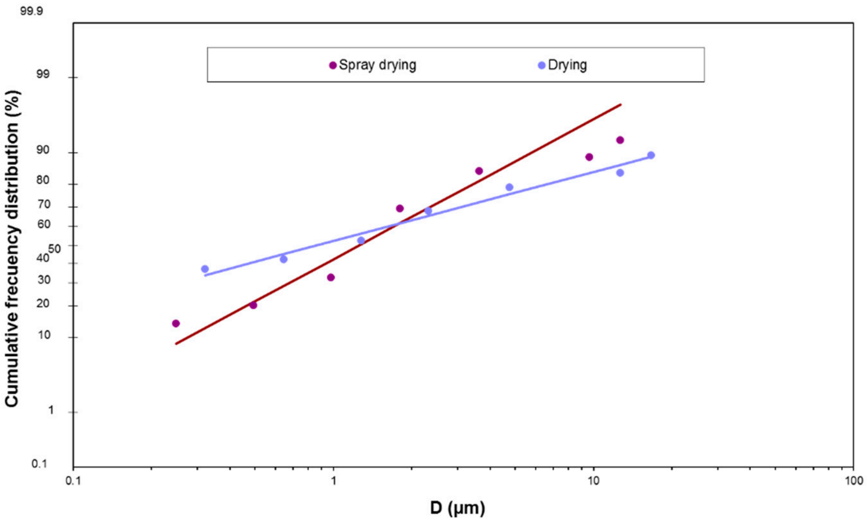

Figure S2. PSD of emissions generated in medium temperature processes

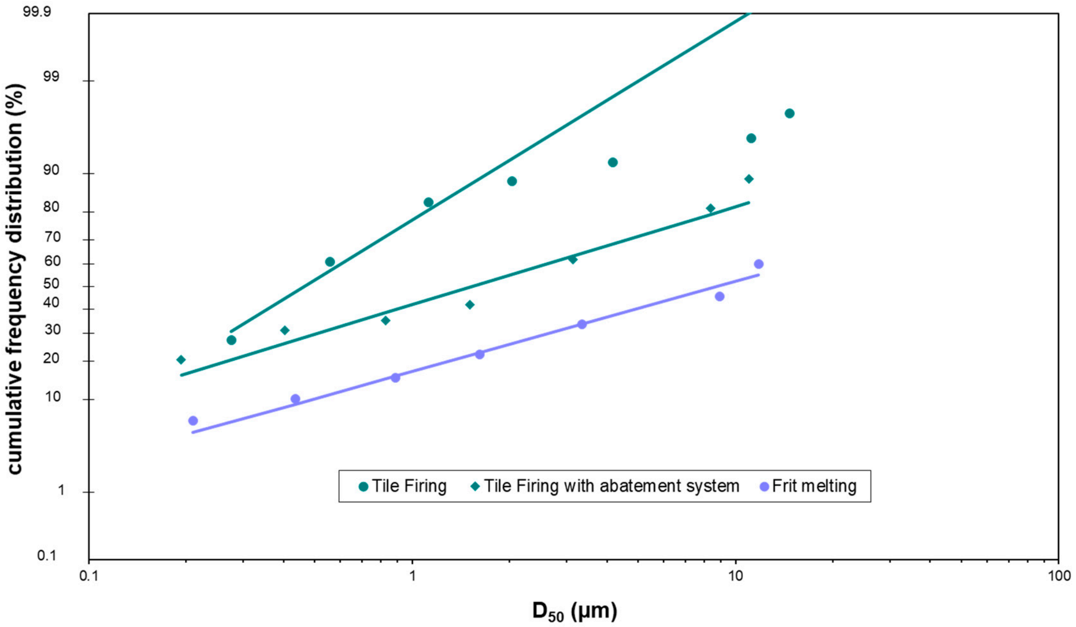

Figure S3. PSD of emissions generated in high temperature processes

Table S1. Individual samplings of ambient temperature processes

Type of process	Process stage	Treatment system	T_{gases} (°C)	C_{TI} (mg/Nm³)	Parameters of the log-normal adjustment D_g(μm)	σ_g	R^2	w_{10} (%)	$w_{2.5}$ (%)	w_1 (%)
Ambient temperature process	Milling	Fabric filter	15 - 30	<5	3.5	8.5	0.9974	68.7	43.7	27.9
				<5	1.2	16.9	0.9827	77.7	60.7	47.9
				<5	1.0	7.1	0.9279	88.3	68.5	50.6
				<5	4.3	9.6	0.9759	64.6	40.6	25.6
	Pressing	Before treatment	15 - 30	109±33	25.0	3.1	0.9981	21.0	2.1	0.2
		Fabric filter		<5	4.7	3.1	0.9532	75.3	28.9	8.5
	Glazing	Before treatment	15 - 40	233±70	17.2	4.9	0.9903	36.7	11.3	3.7
				27±9	5.5	4.0	0.9519	66.8	28.8	11.2
		Fabric filter		<5	1.6	6.5	0.9824	83.8	59.6	40.2
				<5	8.0	3.5	0.9901	57.0	17.7	4.9
				<5	2.7	4.0	0.9840	82.8	47.8	23.7

Table S2. Individual samplings of medium and high temperature processes

Type of process	Process stage	Treatment system	T_{gases} (°C)	C_{TI} (mg/Nm^3)	Parameters of the log-normal adjustment			w_{10} (%)	$w_{2.5}$ (%)	w_1 (%)
					**D_g(μm)**	**σ_g**	**R^2**			
Medium high temperature processes	Drying	No apply	110-120	<5	0.9	11.0	0.9799	84.5	66.9	52.2
	Spray drying	Cyclone	80	>1000	1.8/20.5*	2.0/1.1*	0.9991	73.4	41.3	21.8
		Wet scrubber	60-65	<5	1.2	9.3	0.9151	83.0	63.0	46.8
				<5	4.5	5.8	0.9953	67.8	37.0	19.7
				99±30	1.6	1.7	0.9748	99.9	81.8	19.0
				52±16	0.7	4.9	0.9729	95.4	79.3	59.4
	Firing	Before treatment	160-210	14±4	0.6	2.2	0.9967	99.9	97.1	77.0
				8±2	0.4	4.2	0.9197	98.9	90.6	74.9
		Fabric filter	140-160	<5	1.7	8.3	0.9405	80.2	57.7	40.6
				<5	1.5	7.6	0.9721	83.0	60.6	42.8
	Frits melting	Before treatment	220-300	734±299	1.8 / 21.0*	2.6 / 1.1*	0.9997	77.5	51.1	21.2
				223±67	0.7 / 11.7*	2.5 / 1.3*	1.0000	76.3	62.9	45.7
				287±86	0.3 / 21.5*	2.5 / 1.3*	0.9994	69.4	68.7	62.9
		Fabric filter	110-210	<5	1.4	7.8	0.9808	83.1	61.1	43.5
		Electrostatic precipitator		<5	1.0	6.9	0.9724	88.0	67.5	49.2

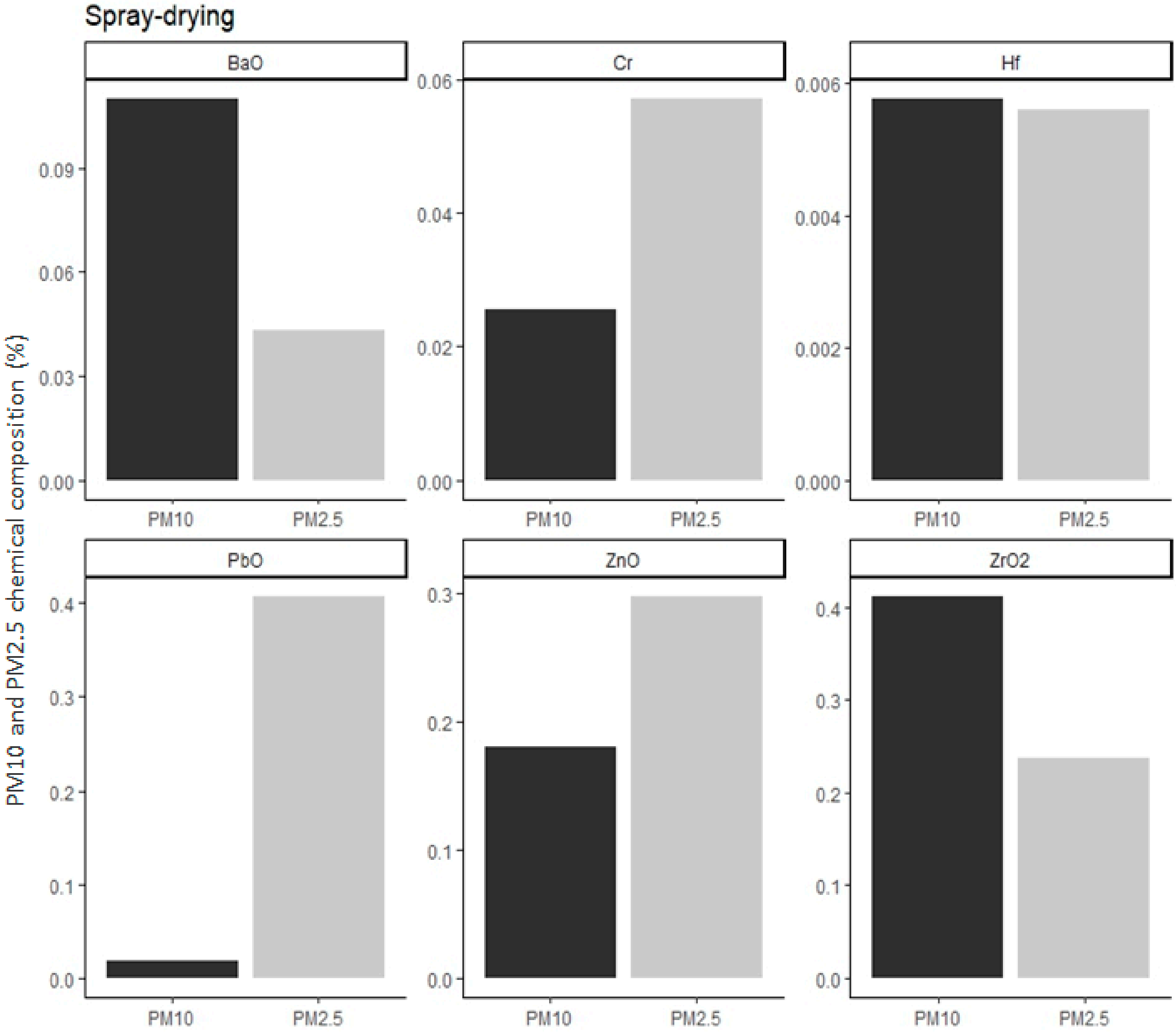

Figure S4. PM10 and PM2.5 composition of spray-drying emissions

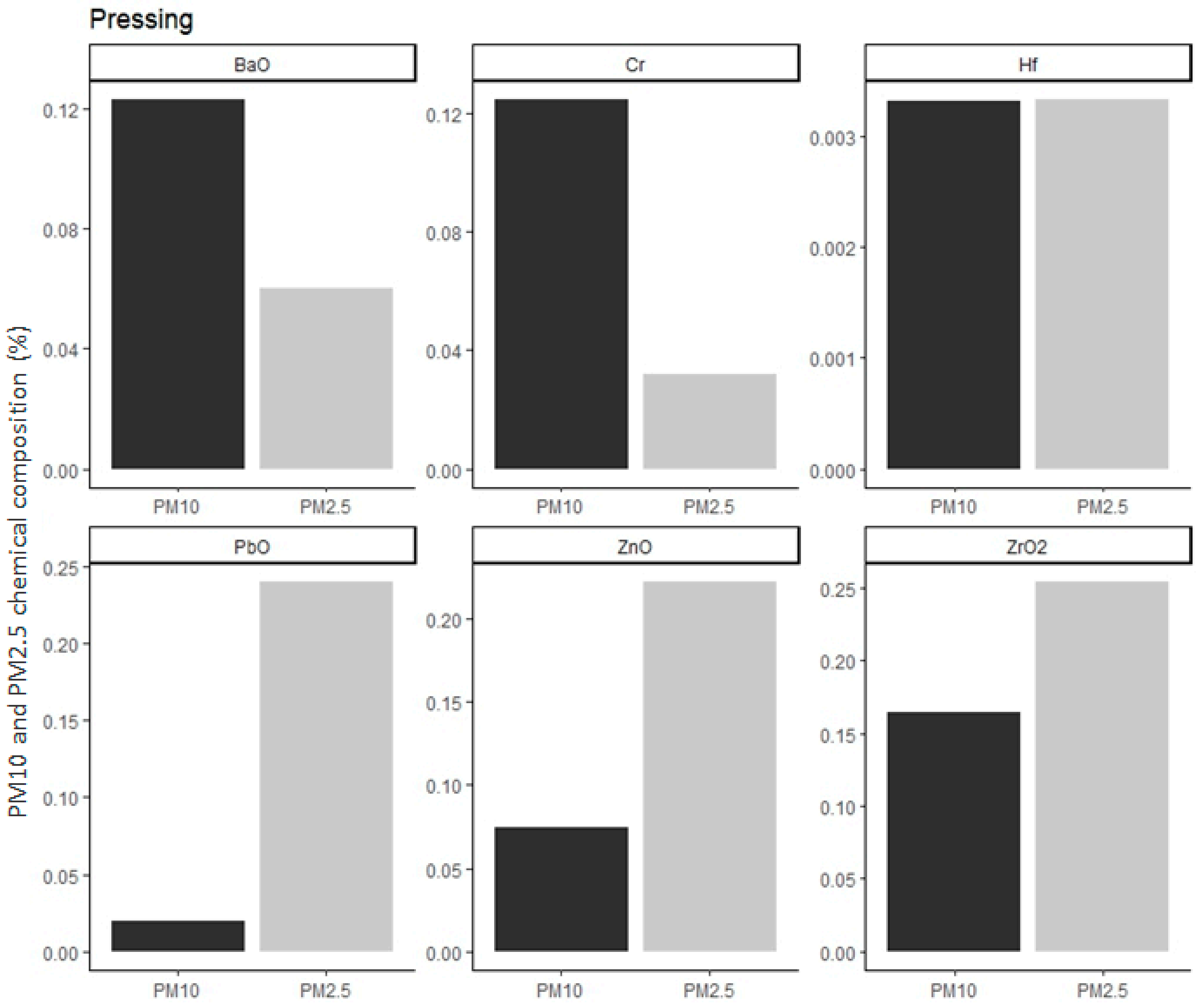

Figure S5. PM10 and PM2.5 composition of pressing

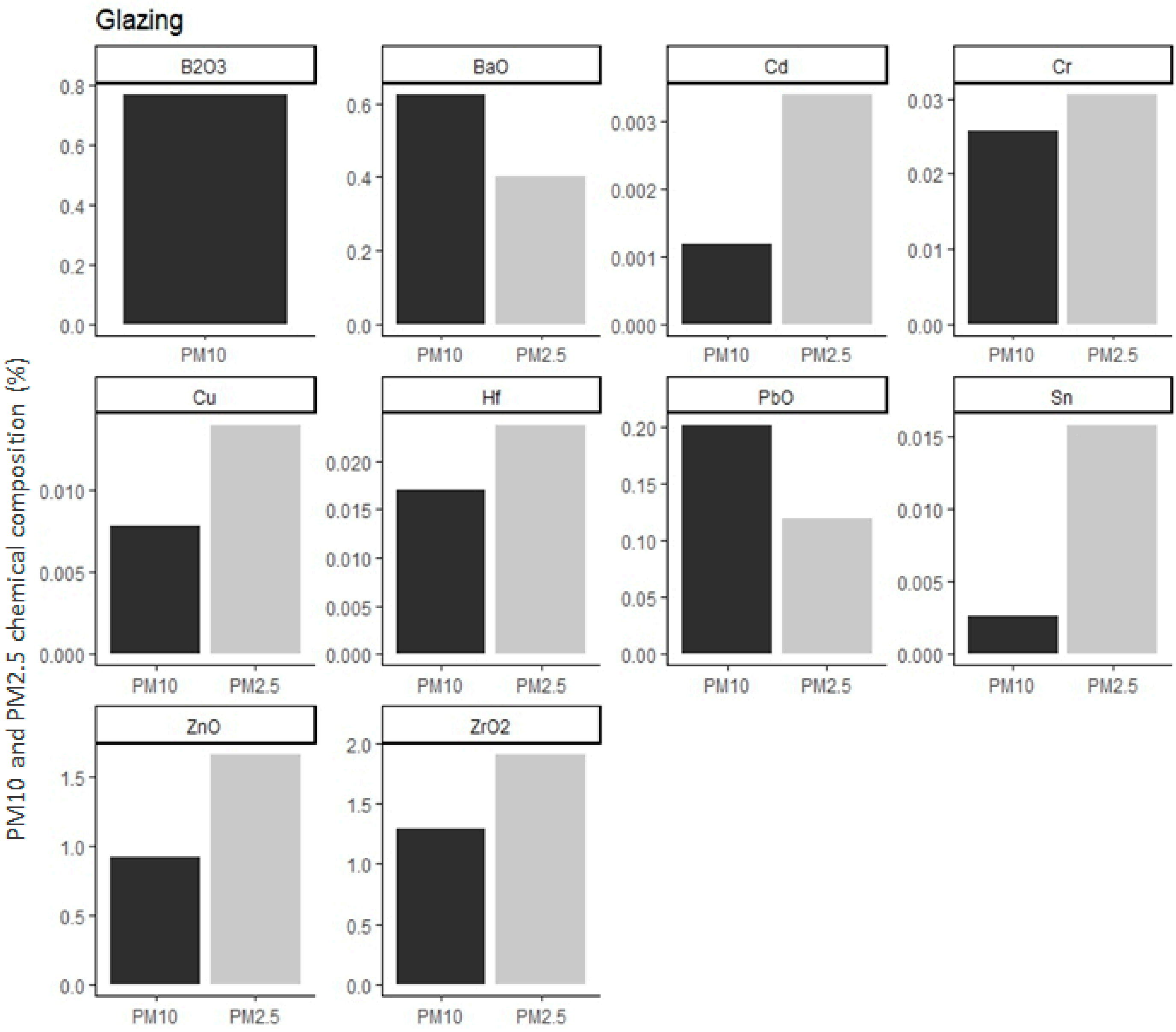

Figure S6. PM10 and PM2.5 composition of glazing

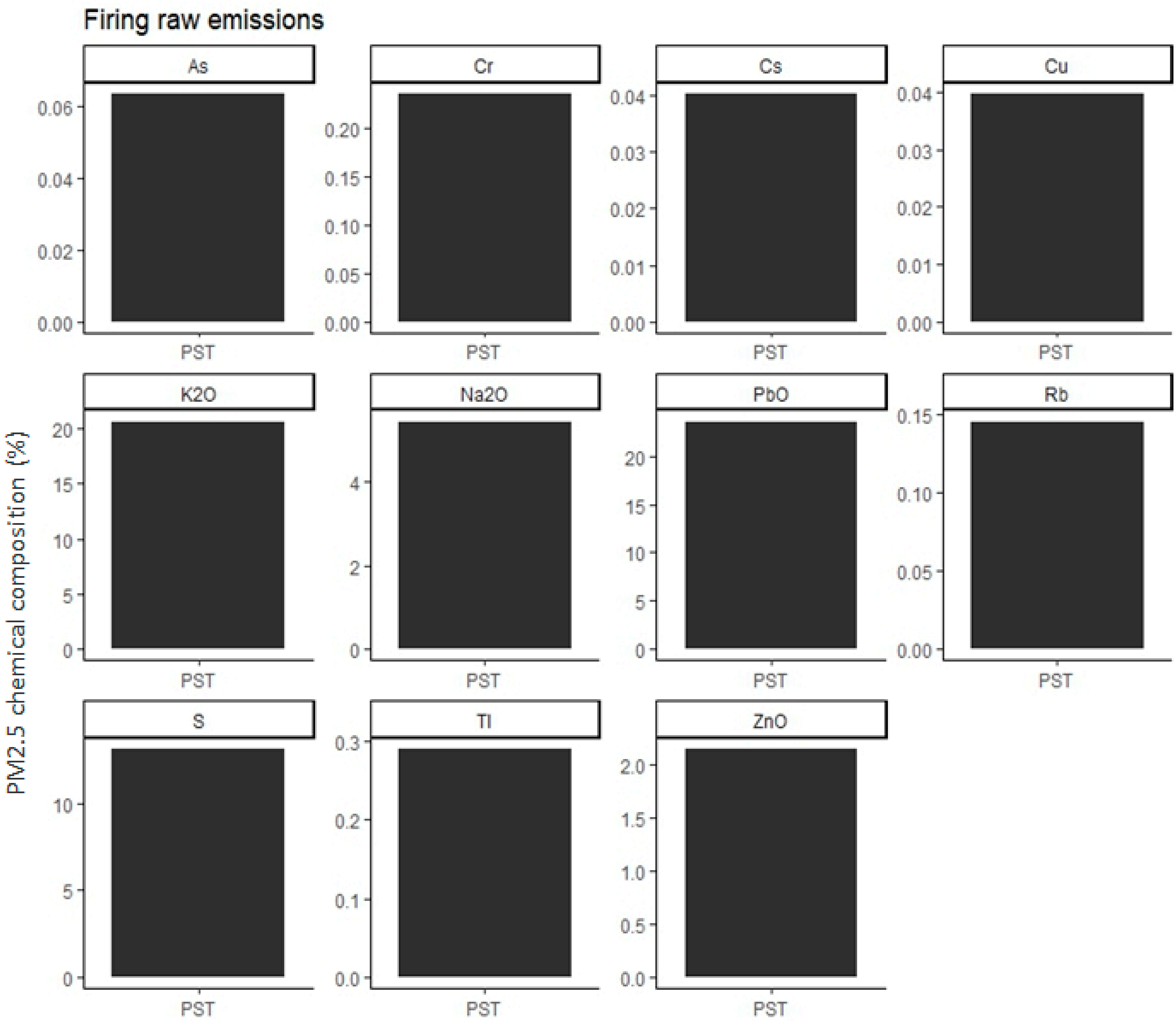

Figure S7. PM10 and PM2.5 composition of emissions generated during firing (raw emissions). Only TSP was analysed because PM2.5 represents more than 90% of TSP.

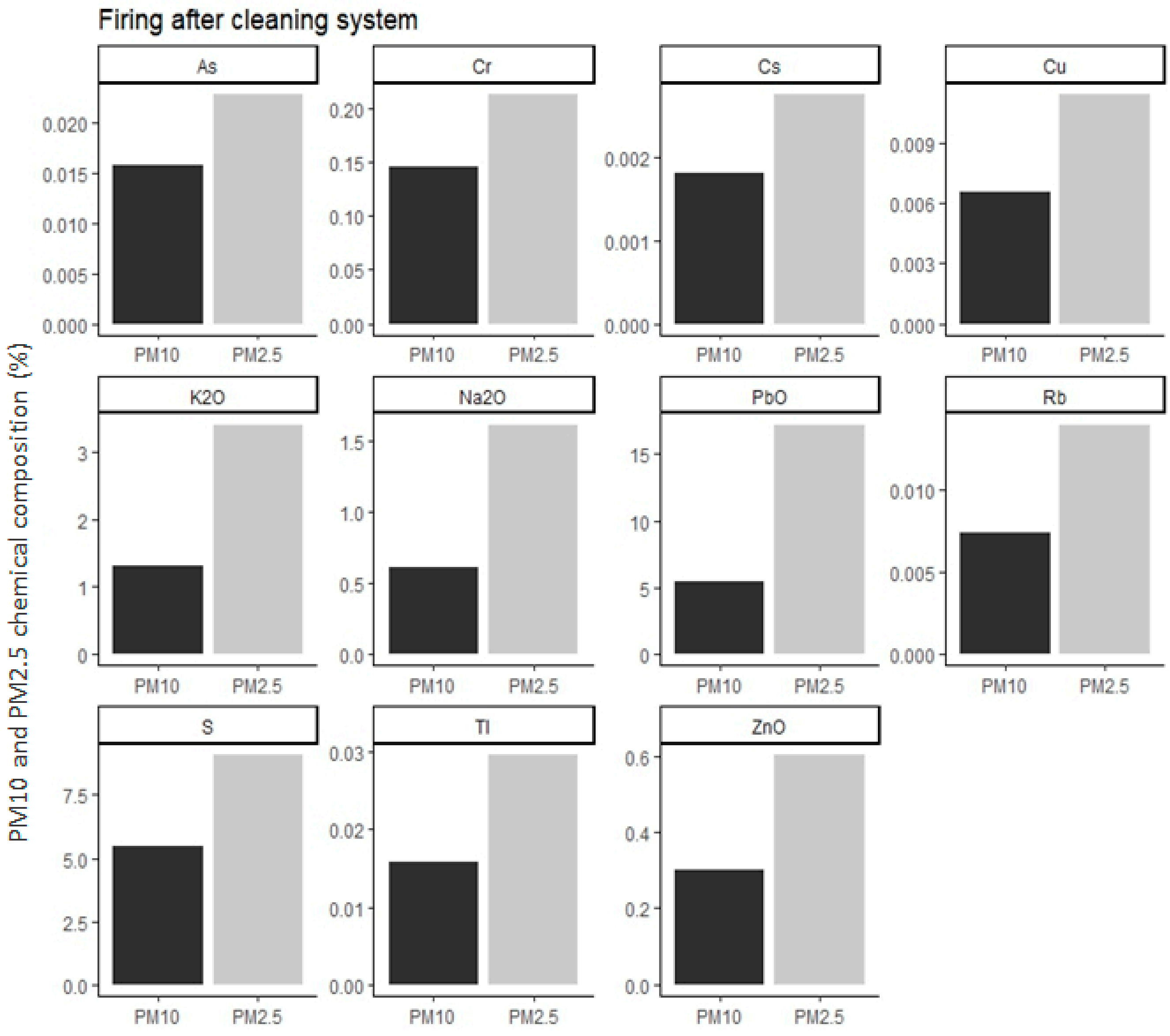

Figure S8. PM10 and PM2.5 composition of emissions generated during firing (after cleaning system).

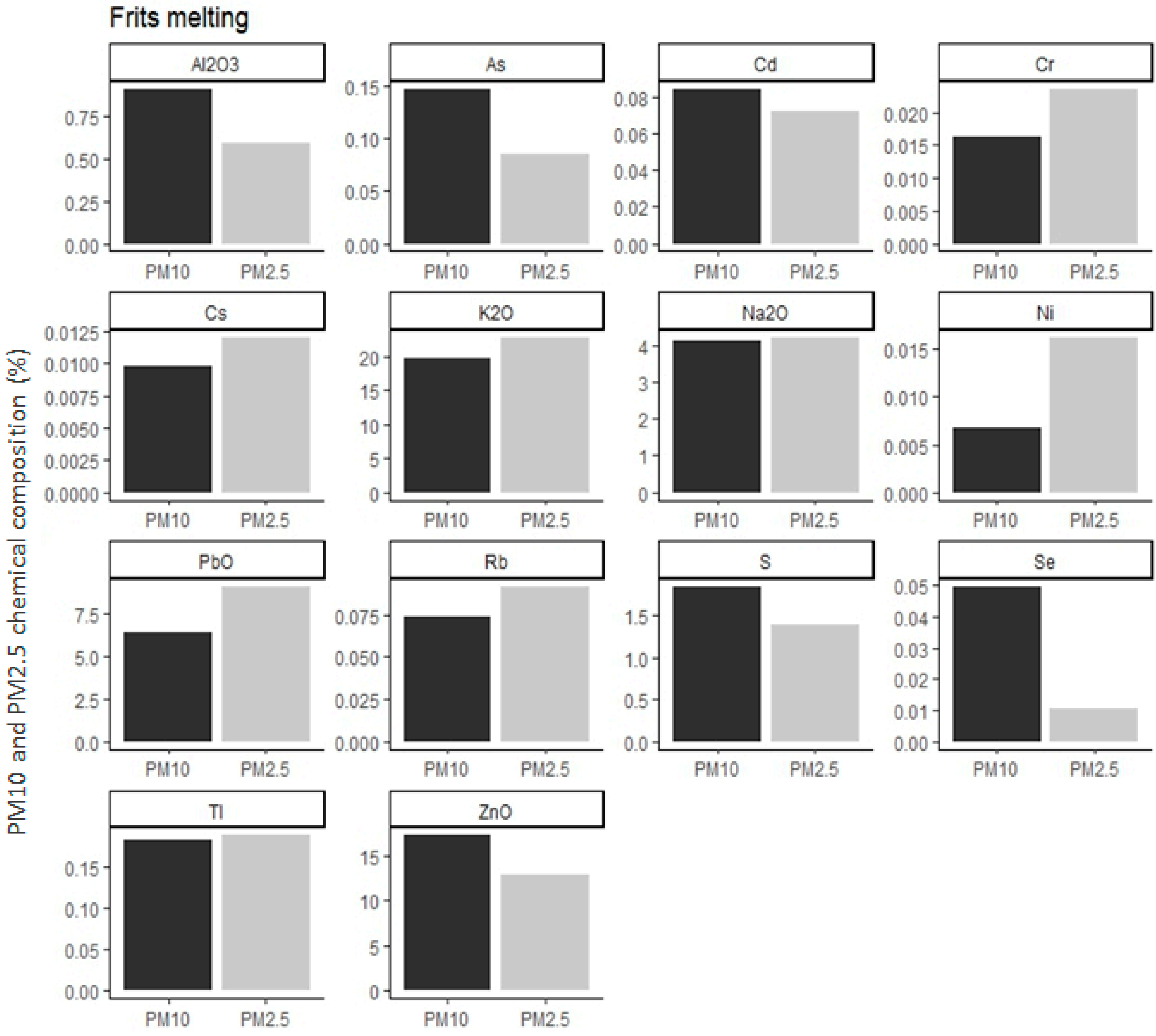

Figure S9. PM10 and PM2.5 composition of f emissions generated during frit melting
